# Outcomes in patients with lacrimal gland carcinoma treated with definitive radiotherapy or eye-sparing surgery followed by adjuvant radiotherapy

**DOI:** 10.1186/s13014-020-01601-8

**Published:** 2020-06-22

**Authors:** Yun-Hsuan Lin, Shih-Ming Huang, Wing-Keen Yap, Ju-Wen Yang, Ling Yeung, Din-Li Tsan, Joseph Tung-Chieh Chang, Lung-Chien Chen

**Affiliations:** 1 Department of Electro-Optical Engineering, National Taipei University of Technology, 1, Sec. 3, Chung-Hsiao E. Rd, Taipei, 10608 Taiwan, ROC; 2 Department of Ophthalmology, Chang Gung Memorial Hospital, Keelung, Taiwan; 3 College of Medicine, Chang Gung University, Taoyuan, Taiwan; 4 Department of Radiation Oncology, Chang Gung Memorial Hospital, Keelung, Taiwan; 5 Department of Radiation Oncology, Chang Gung Memorial Hospital, Linkou, Taiwan

**Keywords:** Lacrimal gland carcinoma, Orbital tumors, Radiotherapy, Orbital exenteration

## Abstract

**Background:**

The optimal treatment for lacrimal gland cancer remains unclear. Eye-preserving surgery, as opposed to exenteration, followed by adjuvant radiotherapy (RT), has recently been reported to deliver satisfactory outcomes, but evidence is sparse. The aim of the present study was to evaluate outcomes in patients with lacrimal gland cancer treated at two tertiary medical centers.

**Methods:**

We retrospectively examined data from patients with lacrimal gland cancer who had received eye-preserving surgical treatment followed by adjuvant RT with or without chemotherapy, or (if the tumor was inoperable) needle biopsy with definitive RT with or without chemotherapy. Baseline clinical and pathological characteristics were considered. Outcomes of interest included post-treatment complications, overall survival (OS), locoregional progression-free survival (LPFS), and distant metastasis-free survival (DMFS).

**Results:**

Eighteen patients were included. Two-year OS, LPFS, and DMFS rates were 69.0, 76.7, and 71.4%, respectively. Patients with early-stage (T1–T2) lacrimal gland cancer had significantly better outcomes than those with advanced-stage disease (T3–T4). Two-year OS, LPFS, and DMFS rates were each 100% in patients with disease stages T1–T2, and 37.5, 50, and 37.5%, respectively, in those with disease stages T3–T4 (*P* < 0.05). Orbital complications were well tolerated.

**Conclusions:**

Eye-sparing surgery with adjuvant RT can achieve satisfactory results in patients with T1–T2 lacrimal gland carcinoma. Disease stage T3 and above was associated with poor outcomes even with post-operative RT, likely due to distant metastasis. Adding neoadjuvant chemotherapy or adjuvant chemotherapy to current treatment strategies might be a suitable choice for this group of patients.

## Precis

Eye-sparing surgery with adjuvant radiotherapy can achieve satisfactory results in patients with T1–2 lacrimal gland carcinoma. Disease stage T3 and above was associated with poor outcomes even with post-operative RT, likely due to distant metastasis.

## Introduction

Lacrimal gland tumors are among the rarest types of head and neck cancers, accounting for approximately 10% of all orbital tumors [1]. Previous studies have estimated annual incidence for these tumors at 0.19–1 per 1,000,000 people [1–3]. Epithelial lesions, including benign pleomorphic adenoma and malignant tumors, account for 20–30% of all lacrimal gland tumors. Malignant epithelial tumors include adenoid cystic carcinoma (ACC) (60%), carcinoma ex pleomorphic adenoma or adenocarcinoma (20–30%), and mucoepidermoid carcinoma (5%) [1, 4, 5]. In contrast, non-epithelial lesions, such as inflammation, lymphoid tumors, plasmacytoma, histiocytoma, lipoma, and hemangioma account for 70–80% of all lacrimal gland tumors [2, 4, 6–8].

Lacrimal gland carcinomas are associated with poor local control and significant morbidity and mortality rates [9]. To date, no guidelines on standard treatment for lacrimal gland carcinomas have been developed due to the rarity of these diseases, which are conventionally treated with orbital exenteration followed by radiotherapy (RT) [5, 10]. However, locoregional recurrence, distant relapse, and cancer-related mortality risks are high after orbital exenteration [5, 11, 12]. Thus, eye-preserving surgery followed by adjuvant RT has recently gained popularity, but the optimal approaches to surgery and RT in these patients remain subject to debate [5, 9, 13, 14].

In this study, we examined clinical outcomes associated with either eye-preserving surgery followed by adjuvant RT, or needle biopsy followed by definitive RT, in a cohort of 18 patients with lacrimal gland carcinoma. Clinical outcomes such as local control, survival, and ocular complications were considered. The clinical and pathological factors that affected the outcomes were also examined.

## Materials and methods

This study protocol was reviewed and approved by the institutional review board of Chang Gung Memorial Hospital (Reference No.: 202000138B0). All data were stored in the hospital database and extracted for research. The participants’ informed consent requirement was waived due to the retrospective nature of this study.

### Study population

This retrospective study included patients with lacrimal gland cancer who underwent either eye-preserving surgical treatment followed by adjuvant RT with or without chemotherapy, or (in cases involving an inoperable tumor), needle biopsy followed by definitive RT with or without chemotherapy. Patients diagnosed with recurrent tumors at their first visit, and patients diagnosed with lacrimal duct cancer or other type of orbital cancer, were excluded. Disease stage was reviewed by a radiologist and ophthalmologist based on the American Joint Committee on Cancer (AJCC) staging system, eighth edition. All patient records included detailed pathological reports with data on cell type, margin status, perineural invasion (PNI), and lymphovascular invasion (LVSI), if the tumor had undergone resection.

### Treatment

Radiotherapy was recommended for patients at high risk of recurrence, including those with an advanced stage lacrimal gland cancer and/or multiple unfavorable pathological factors such as bony structure invasion, positive surgical margins, LVSI, or PNI. RT was delivered in the form of three-dimensional conformal RT, intensity modulated RT, volumetric modulated arc therapy, or proton beam therapy. The RT field included the tumor bed and the residual gross tumor volume. The median prescribed dose for adjuvant RT was 50–60 Gy and 66–70 Gy for the definitive setting, with 1.8–2 Gy daily fractions administered over 5–7 weeks. Whether or not chemotherapy was given was at the attending physician’s discretion. The regimens used were platinum-based chemotherapy.

### Statistical analyses

Overall survival (OS) was defined as the time from the date of surgical treatment or biopsy until death or the last follow-up appointment. Local progression-free survival (LPFS) was defined as the time between the date of surgical treatment or biopsy and the detection of local tumor progression. Data on the first site(s) of distant metastasis were collected, and distant metastasis-free survival (DMFS) was determined. The Kaplan–Meier method was used to estimate the OS, LPFS, and DMFS rates, while the statistical significance of between-group differences in clinical characteristics (including sex, age, RT dose, cell types, and pathological features) and OS, LPFS, DMFS was determined using the log-rank test. All statistical analyses were performed using SPSS v. 23.0 (IBM Corp., New York, NY; formerly SPSS Inc., Chicago, IL). All *P*-values were two-sided, and a *P*-value < 0.05 was considered statistically significant.

### Post-therapy surveillance

Follow-up in the form of radio–oncologic and ophthalmic clinic appointments was arranged every 3 months during the first 2 years, every 4–6 months during the third and fourth year, and every 6–12 months thereafter. Imaging examination was performed at specific intervals: chest radiography every 3 months, and computed tomography or orbital magnetic resonance imaging every 3–6 months, until symptoms indicating recurrence were noted.

### Ocular complication evaluation

Follow-up in the form of ophthalmic clinic appointments was arranged every 3 months during the first 2 years and every 6–12 months thereafter. Detailed ocular examination, including visual acuity, intraocular pressure, slit lamp examination, and fundus examination was performed at every ophthalmologic visit. Medical or surgical treatment was administered if an ocular complication was noted.

## Results

### Patient characteristics

During 2000–2018, a total of 18 patients from the Linkou and Keelung branches of Chang Gung Memorial Hospital were included in the present study. The median follow-up time was 39 months (range 5–161 months) for the patients who were alive at the end of the study period, and 26 months (range 5–161 months) for the whole cohort. The patients’ demographic and clinical characteristics are shown in Table 1 and Table 2. Fifteen patients (83.3%) underwent eye-sparing surgery and the remaining patients had needle biopsy alone due to inoperable tumor invading brain tissue or extending to the infratemporal fossa. ACC was found in nine, carcinoma ex pleomorphic adenoma in seven, and poorly differentiated adenocarcinoma in two patients. In five patients (27.8%), negative margins were achieved during surgery. Six patients (33.3%) had pathologically confirmed LVSI and eight patients (44.4%) had pathologically confirmed PNI. Patients who underwent eye-sparing surgery also received adjuvant RT or concurrent platinum-based chemoradiotherapy (CCRT) due to the presence of risk factors, such as positive surgical margin, confirmed LVSI or PNI, or initial advanced tumor size and invasion status. Three patients who underwent needle biopsy received definitive RT or CCRT, also platinum-based.

**Table 1:** Baseline characteristics of patients with lacrimal gland carcinoma

Total (*n* = 18)	Patient number	Percentage (%)
Median age (years)	54 (20–85)	
Sex		
Male	8	44.4
Female	10	55.6
T stage		
1	1	5.6
2	9	50.0
3	3	16.7
4	5	27.7
Types of surgery		
Excision	15	83.3
Needle biopsy	3	16.7
RT treatment		
Adjuvant RT alone	13	72.2
Adjuvant CCRT	2	11.1
Definitive RT	2	11.1
Definitive CCRT	1	5.6
RT type		
3DCRT	2	11.1
IMRT	14	77.8
Proton	2	11.1
RT dose		
< 6600 cGy	9	50.0
≥ 6600 cGy	9	50.0
Cell type		
Adenoid cystic carcinoma	9	50.0
Carcinoma ex pleomorphic adenoma	7	38.9
Poorly differentiated adenocarcinoma	2	11.1
Margin		
positive	13	72.2
negative	5	27.8
LVSI		
Positive	6	33.3
Negative	12	66.7
PNI		
Positive	8	44.4
Negative	10	55.6

*RT* radiotherapy, *CCRT* concurrent chemoradiotherapy, *3DCRT* 3-dimensional conformal radiotherapy, *IMRT* intensity-modulated radiotherapy, *cGy* centigray, *LVSI* lymphovascular invasion, *PNI* perineural invasion.

**Table 2:** Detailed patient, tumor, treatment characteristics and outcomes

Patient	Age	Gender	Cell type	AJCC stage	laterality	Surgery type	Margin	PNI	LVSI	Adjuvant treatment	Post treatment status	Time to local progression (months)	Time to distant metastasis (months)	Metastasis site	Total follow up time (months)	Final status
1	45	Female	CEPA	cT1aN0M0	L	Excision	+	–	–	RT alone	CR	–	–		161	alive
2	20	Female	ACC	cT2aN0M0	L	excision	–	+	–	RT alone	CR	–	–		28	dead
3	58	Female	ACC	cT2aN0M0	L	excision	+	–	–	RT alone	CR	–	–		20	alive
4	55	Male	CEPA	cT2aN0M0	R	excision	+	–	+	RT alone	CR	–	–		39	alive
5	77	Male	CEPA	cT2aN0M0	R	excision	+	–	–	RT alone	CR	–	–		26	alive
6	53	Female	CEPA	cT2aN0M0	L	excision	–	+	+	RT alone (proton)	CR	–	–		5	alive
7	45	Male	ACC	cT2bN0M0	L	excision	+	–	–	CCRT (proton + photon)	CR	–	–		14	alive
8	35	Male	ACC	cT2cN0M0	L	excision	+	+	+	RT alone	CR	–	–		158	alive
9	43	Female	ACC	cT2cN0M0	R	excision	+	+	–	RT alone	CR	–	26	mediastinal LNs, liver, bone, lungs	26	dead with disease
10	53	Female	ACC	cT2cN0M0	R	excision	+	+	+	RT alone	CR	–	–		40	alive
11	85	Male	ACC	cT3cN0M0	L	needle biopsy	–	–	–	RT alone	PR	10	34	lungs	43	alive with disease
12	62	Male	CEPA	cT3cN0M0	L	excision	+	–	+	RT alone	CR	50	51	lungs	92	dead with disease
13	80	Male	CEPA	cT3cN0M0	R	needle biopsy	–	–	–	RT alone	CR	–	–		17	dead
14	36	Female	ACC	cT4bN0M0	L	excision	+	+	–	RT alone	CR	10	6	liver	16	dead with disease
15	30	Female	ACC	cT4bN0M0	L	excision	+	+	–	RT alone	CR	5	10	bone and lungs	28	dead with disease
16	58	Female	CEPA	cT4bN0M0	R	excision	+	+	+	RT alone	CR	6	5	parotid, neck nodes, lungs	12	dead with disease
17	60	Male	PD adeno	cT4cN0M0	L	needle biopsy	–	–	–	CCRT	PR	–	5	bone	20	dead with disease
18	71	Female	PD adeno	cT4cN0M0	R	excision	+	–	–	CCRT	CR	–	5	liver and bone	15	dead with disease

*AJCC* the American Joint Committee on Cancer, *PNI* perineural invasion, *LVSI* lymphovascular invasion, *ACC* adenoid cystic carcinoma, *CEPA* carcinoma ex pleomorphic adenoma, *PD adeno* poorly differentiated adenocarcinoma, *L* left, *R* right, *RT* radiotherapy, *CCRT* concurrent chemoradiotherapy, *CR*=, *LN* lymph node.

**Fig. 1:**
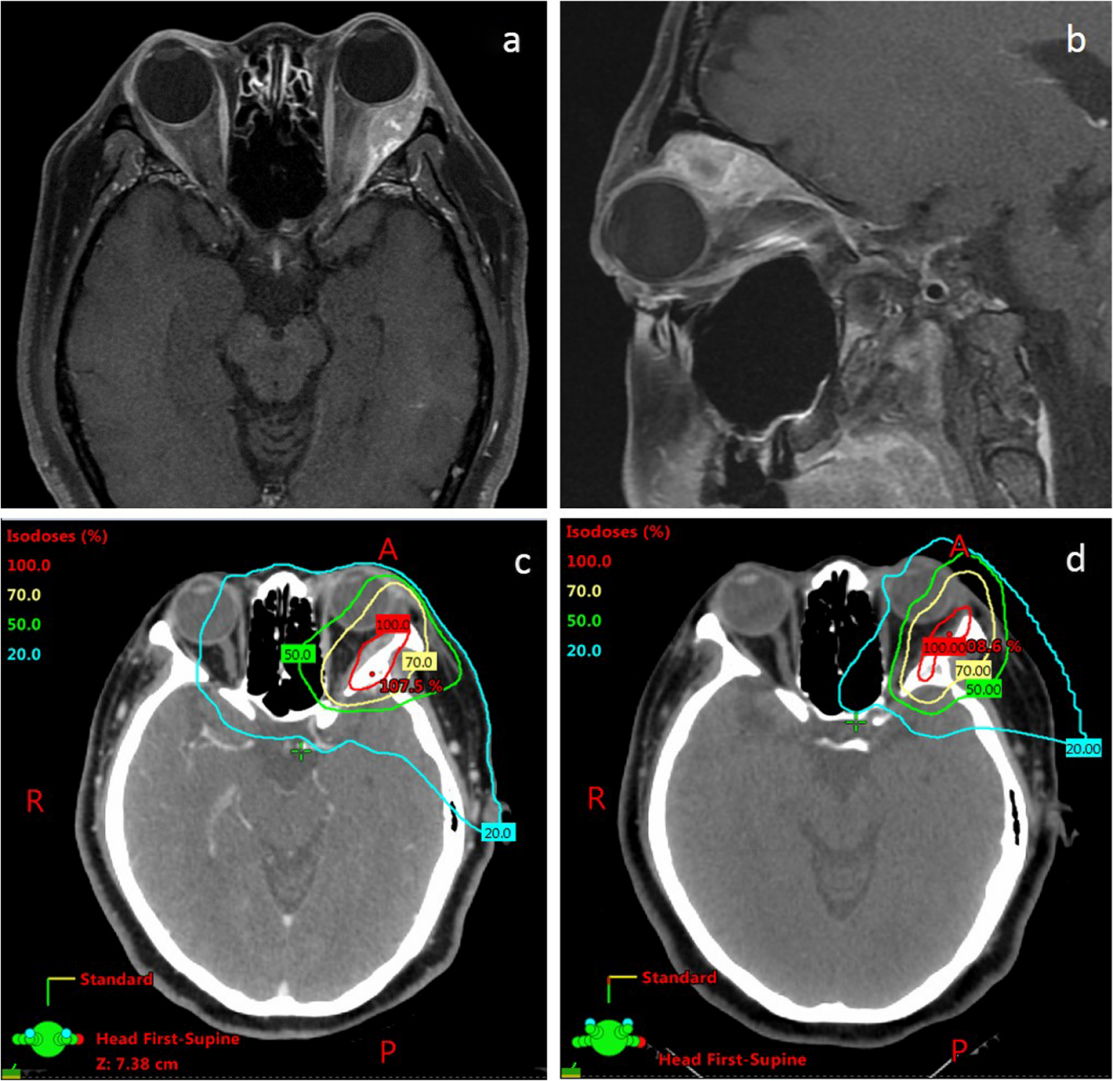
Patient number 7, cT2bN0M0 lacrimal gland cancer, pre-surgery T1-MRI and the photon or proton beam dosimetry of adjuvant radiotherapy. Total RT dose was 70 Gy in 33 fractions. Dose constraints for organs at risk: Brain stem: D_max_ < 54 Gy. Left eye and left optic nerve: D_max_ ≤ 59.5Gy (85% dose). Right eye and right optic nerve: D_max_ ≤ 50 Gy. Optic chiasm: D_max_ < 54 Gy. Right len: D_max_ ≤ 18Gy. No ocular complication was noted during our study period. **a**. Axial view of the tumor. **b**. Sagittal view of the tumor. **c**. Photon beam dosimetry. 1d. Proton beam dosimetry

Regarding RT technique, 2 patients received three-dimensional conformal radiotherapy (3DCRT), 14 received intensity-modulated radiotherapy (IMRT), one received proton beam therapy alone, and one received combined proton–photon RT. The dosimetry comparison between photon plan and proton plan is presented in Fig. 1. The median RT dose for adjuvant RT was 6000 cGy (range 3600–7000 cGy) in 1.8–2 Gy per fraction, and the definitive RT dose was 6000 cGy/25 fractions, 7200 cGy/36 fractions, and 7200 cGy/36 fractions for three patients, respectively.

### Survival analysis

**Fig. 2:**
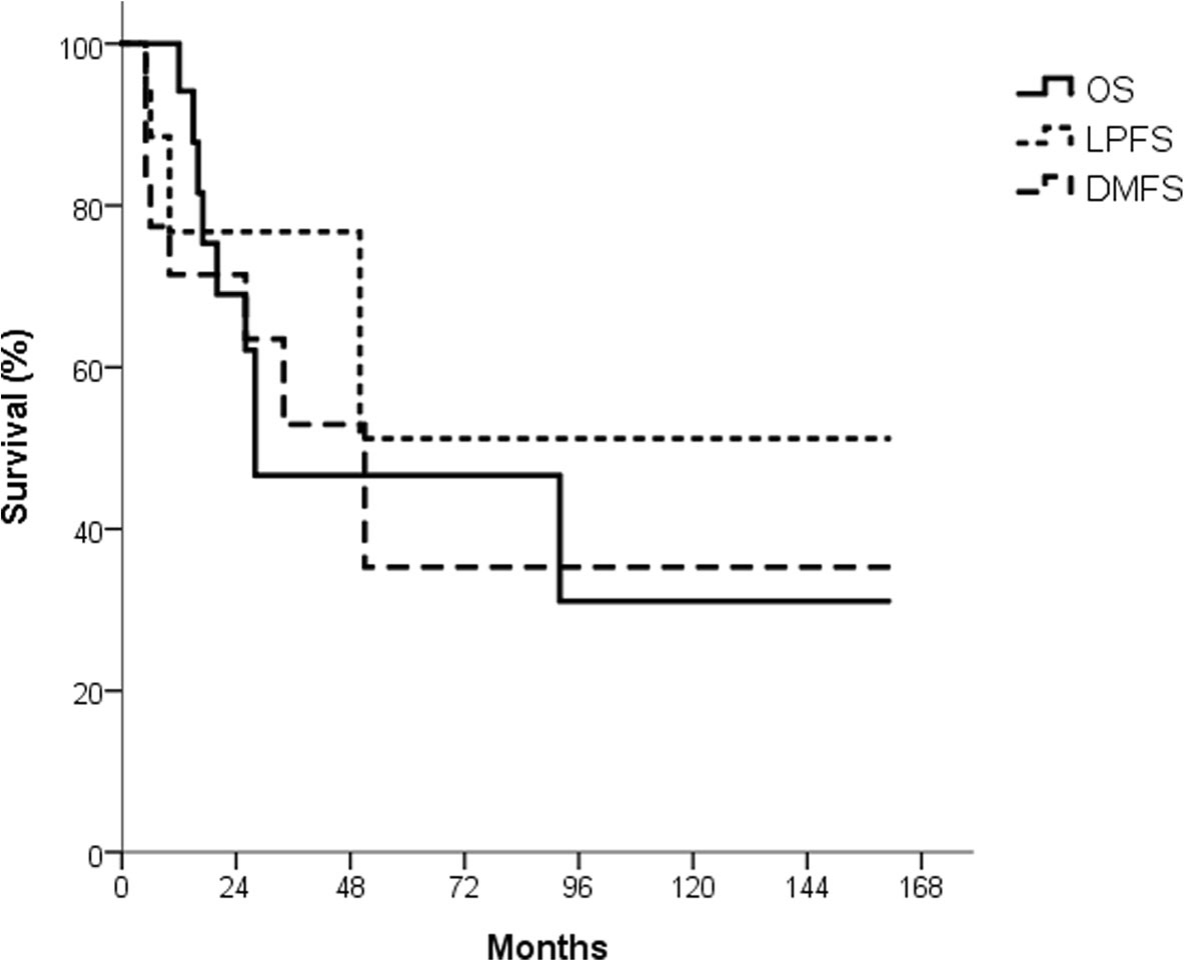
The overall survival (OS), local progression-free survival (LPFS), and distant metastasis-free survival (DMFS) graphs of the whole cohort. For the entire cohort, 2-year OS, LPFS, and DMFS rates were 69.0, 76.7, and 71.4%, respectively

**Fig. 3:**
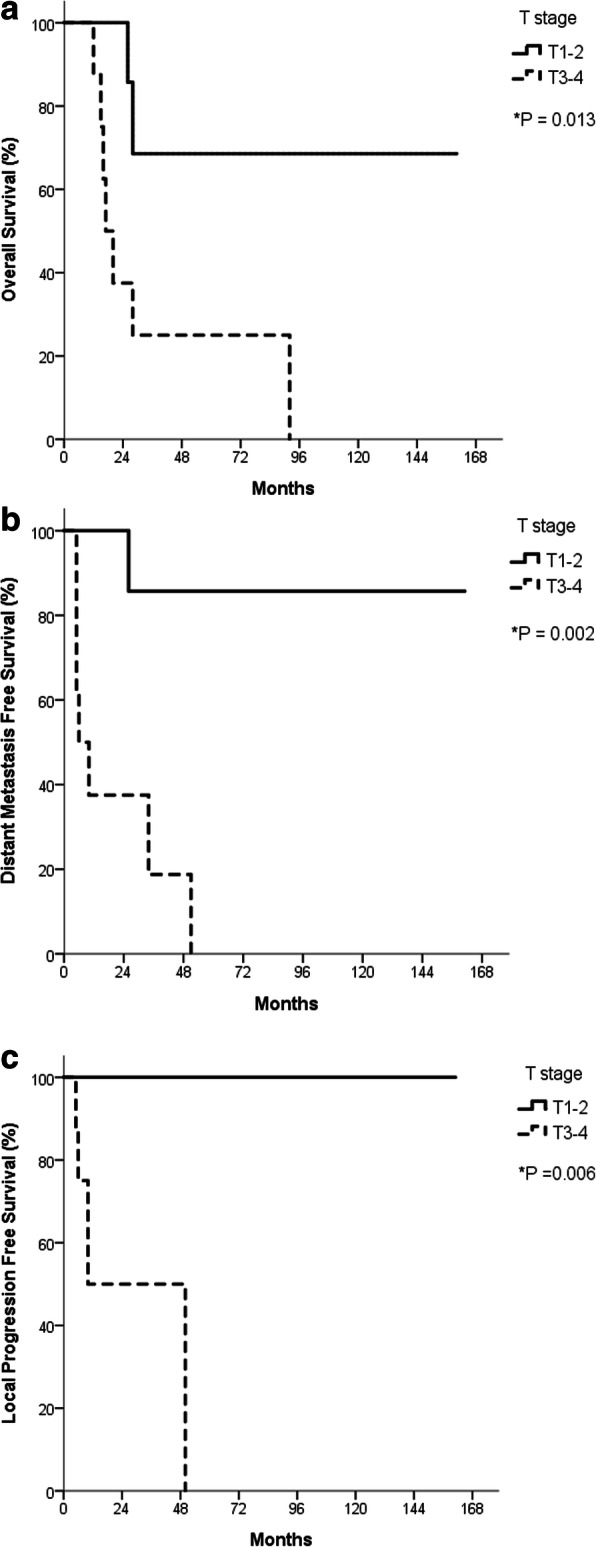
Kaplan-Meier survival curves showing overall survival (**a**), local progression free survival (**b**), and distant-metastasis free survival (**c**) in patients with lacrimal gland carcinoma according to T category as determined using the eighth edition of the American Joint Committee on Cancer staging system. * Log-rank test. (**a**) The 2-year OS rates were 100 and 37.5% in T1–2 and T3–4 groups (*p* = 0.013), with (**b**) the 2-year DMFS rates were 100 and 37.5% (*p* = 0.002), and (**c**) the 2-year LPFS rates of 100 and 50%, respectively (*p* = 0.006)

For the entire cohort, 2-year OS, LPFS, and DMFS rates were 69.0, 76.7, and 71.4%, respectively (Fig. 2). Associations between clinicopathological factors and 2-year OS, LPFS, and DMFS rates are shown in Table 3. Patients with early-stage (T1–T2) lacrimal cancer achieved significantly better 2-year OS, LPFS, and DMFS than those with advanced-stage disease (T3–T4). Two-year OS was 100% in patients with disease stage T1–T2, and 37.5% in those with disease stage T3–T4. Moreover, 2-year LPFS was 100% in patients with disease stage T1–T2 and 50% in those with disease stage T3–T4. Finally, 2-year DMFS was 100% in patients with disease stage T1–T2 and 37.5% in those with disease stage T3–T4 (Fig. 3). Other factors, including sex, age (< 54 yrs. vs. ≥54 yrs), RT dose (< 66 vs. ≥66 Gy), cell types (ACC vs. others), margin (positive vs. negative), PNI (positive vs. negative), and LVSI (positive vs. negative) had no significant impact on OS, LPFS, or DMFS.

**Table 3:** Two-year overall survival, local progression-free survival, and distant-metastasis free survival rates for patients with lacrimal gland carcinoma, including factors affecting survival

	2-Y OS (%)	*P*-value*	2-Y LPFS (%)	*P*-value*	2-Y DMFS (%)	*P*-value*
Sex (male vs. female)	87.5 vs. 67.5	0.533	87.5 vs. 67.5	0.533	87.5 vs. 57.1	0.353
Age (< 54 vs. ≧ 54)	85.7 vs. 55.6	0.529	76.2 vs. 77.8	0.621	75.0 vs. 66.7	0.331
T stage (T1–2 vs. T3–4)	100.0 vs. 37.5	0.013	100.0 vs.50.0	0.006	100.0 vs. 37.5	0.002
RT dose (< 66 vs. ≧66 Gy)	87.5 vs. 50.8	0.114	63.5 vs. 88.9	0.464	75.0 vs. 66.7	0.344
Cell types (ACC vs. others)	87.5 vs. 50.0	0.517	66.7 vs. 87.5	0.581	77.8 vs. 66.7	0.914
Margin (positive vs. negative)	75.5 vs. 50.0	0.43	76.9 vs. 75.0	0.944	69.2 vs. 80.0	0.76
PNI (positive vs. negative)	66.7 vs. 71.4	0.438	58.3 vs. 90.0	0.264	58.3 vs. 80.0	0.583
LVSI (positive vs. negative)	80.0 vs. 75.0	0.879	80.0 vs. 75.0	0.879	83.3 vs. 66.7	0.292

*Log-rank test

*Y* year, *OS* overall survival, *LPFS* local progression free survival, *DMFS* distant-metastasis free survival, *RT* radiotherapy, *ACC* adenoid cystic carcinoma, *PNI* perineural invasion, *LVSI* lymphovascular invasion

### Ocular toxicity analysis

In our study subjects, one patient was lost follow up at the ophthalmic clinic 3 months after surgery and three patients 6 months after surgery. During their follow-up period, no ocular complications were reported. In the remaining 14 patients who were followed up by the ophthalmologist for more than 12 months, the administered treatment was well tolerated. Treatment-related acute and late ocular toxicity effects are shown in Table 4. Ocular toxicities, including punctate keratitis, developed in five patients (27.78%) 1.14–7.9 months after radiotherapy (RT total/fraction dose: 5000–7000/200 cGy). Cataract developed in four patients (22.22%) and occurred 2.13–112.7 months after the RT (RT total/fraction dose: 6000–6600/200 cGy). Dry eye developed in two patients (11.11%) and occurred 6.29–11.05 months after the RT (RT total/fraction dose: 6600/200 cGy). Blepharitits, which developed in two patients (11.11%), occurred 2.06–8.00 months after the treatment (RT total/fraction dose: 6600/200 cGy). Other complications, including trichiasis, acute conjunctivitis, corneal epithelial defect, filamentary keratitis, radiation retinopathy, and vitreous hemorrhage were each reported in one patient (5.56%). The patient with radiation retinopathy received an intravitreous injection of 1.25 mg Avastin (*bevacizumab)* for retinal neovascularization.

**Table 4:** Frequency of ocular complications after radiotherapy reported in the current study and the corresponding radiotherapy dose

Complications	Patient number	Percentage (%)	Time of occurrence (m)	RT total dose (cGy)	RT fraction dose (cGy)
Punctate keratitis	5	27.78	1.14–7.9	5000–7000	200
Cataract	4	22.22	2.13–112.7	6000–6600	200
Dry eye	2	11.11	6.49–11.05	6600	200
Blepharitis	2	11.11	2.06–8.00	6000	200
Trichiasis	1	5.56	16.36	6000	200
Acute conjunctivitis	1	5.56	0.68	5000	250
Corneal epithelial defect	1	5.56	23.38	7200	200
Filamentary keratitis	1	5.56	6.65	7000	200
Radiation retinopathy	1	5.56	74.39	6600	200
Vitreous hemorrhage	1	5.56	10.92	6840	180

*RT* radiotherapy, *cGy* centigray

## Discussion

This study reported outcomes in patients with lacrimal gland carcinoma who underwent either needle biopsy followed by definitive radiotherapy with or without chemotherapy due to inoperable tumor, or eye-preserving surgical treatment followed by adjuvant radiotherapy with or without chemotherapy. In the present cohort, 2-year OS, LPFS, and DMFS rates were 69.0, 76.7, and 71.4%, respectively. These outcomes were inferior to those reported in previously, which was probably due to differences in patient characteristics. Esmaeli et al. [15] studied the eye-sparing approach to treating lacrimal gland carcinoma in 11 patients during 2007–2014. All 11 patients were disease-free at the last follow-up visit after the eye-sparing surgery, with a median follow-up time of 33 months (range, 14–64 months). A significant proportion of patients recruited by Esmaeli et al. had early-stage disease (one patient with T1, six patients with T2, one with T3, and three with T4). In addition, Han et al. [16] reported outcomes following eye-sparing surgery and adjuvant radiotherapy in 10 patients with adenoid cystic carcinoma of the lacrimal gland, treated during 1998–2012 (one patient with T1, seven with T2, and two with T3 stage disease), at a median follow-up time of 89.5 months (range 37–217 months). Reported OS was 90%, with a single patient who died 58 months after surgery. Moreover, DMFS was 100%, while local recurrence occurred in one patient. In our series, almost half of the patients had advanced-stage lacrimal gland carcinoma (three patients with T3 [16.7%], and five patients with T4 [27.7%]). In addition, the majority of patients were at high risk of locoregional or distant recurrence, with a positive surgical margin in 72.2% of the patients, and confirmed LVSI and PNI in 33.3 and 44.4% of patients, respectively. The differences in patient baseline characteristics may explain the relatively lower OS, LPFS, and DMFS rates reported in our study.

The present study is among a small number that have examined factors affecting outcomes in patients with lacrimal gland carcinoma, which is an aim that is difficult to achieve due to the rarity of this disease. In a previous report by Woo et al., the only tumor characteristic associated with outcome was T stage. In the same study, adjuvant radiotherapy was strongly associated with a better 5-year recurrence-free survival rate [17]. Friedrich and Bleckmann also found that lower T stage was associated with a better prognosis [18]. Among factors reported as negatively affecting patient outcomes were older age, basaloid or solid pattern ACC, perineural invasion, and the presence of a macroscopic tumor on imaging scans before RT. [12, 19–22] In the present study, among patients who had received adjuvant radiotherapy, T stage and tumor size emerged as the most important factors that determined patient outcome. Specifically, patients with tumor at stage T1–2 treated with eye-sparing surgery followed by adjuvant radiotherapy achieved 100% 2-year LPFS, DMFS, and OS rates. However, tumor stage T3 and higher was associated with poor outcomes even when treated with surgical resection followed by adjuvant RT, which occurred most likely due to distant metastasis. Adding neoadjuvant chemotherapy or adjuvant chemotherapy to current treatment strategies might be a suitable choice for this group of patients to improve the control of distant disease.

Proton therapy or even heavy ion therapy applied to lacrimal gland cancer is considered promising for reducing low dose delivered outside the treated fields [3]. We have observed better dosimetry associated with the proton plan compared to the photon plan. At a high dose (> 70% dose), proton beam dosimetry is equivalent to photon therapy; however, low-dose distribution is better for proton therapy, whereby the 20% isodose line can spare the opposite eye. Ensuring low dose spread to the nearby healthy tissue is critical in certain cases. Lower dose delivered to the regional organ at risk can reduce the likelihood of secondary malignancy and long-term complications, and therefore should be considered for patients with early-stage lacrimal gland cancer who are likely to achieve good local control and long-term survival after the treatment.

Several ocular toxicities were recorded among our patients. These complications were expected as the radiation doses used were mostly above the dose that is well tolerated by ocular structures other than the sclera. Sensitivity to radiation of the ocular structures has been shown to vary [23–25]. The lens has been reported as the most radiosensitive ocular structure, with a minimum cataractogenic dose of 5.5 Gy. The lacrimal gland, cornea, and conjunctiva can each tolerate up to 50 Gy of radiation. The retina tolerates doses > 55 Gy. The sclera is the most resistant ocular structure and is believed to tolerate doses > 1000 Gy. The majority of ocular complications reported in the present study were effectively managed by ophthalmologists. Only one patient received a punctal plug due to intolerable dry eye and another patient required intravitreous injection of Avastin (*bevacizumab)* at 1.25 mg due to radiation-associated retinopathy with retinal neovascularization. Therefore, we recommend that all patients who have received RT for lacrimal gland carcinoma visit an ophthalmologic clinic regularly.

This study has some limitations. First, this was a retrospective study based on medical records, which makes selection bias inevitable. Moreover, some of the patients did not attend the ophthalmologic clinic as recommended, making it likely that the rate of ocular complications was underestimated. Second, due to the rarity of lacrimal gland cancer, the small sample size meant that the analysis was unlikely to yield statistically significant results. Finally, the patients receiving proton treatment were only followed for 1–2 years, as our hospital began proton beam therapy in 2017. A longer follow-up period is required to evaluate the effects of proton therapy on patient survival.

In conclusion, eye-sparing surgery with adjuvant RT can achieve satisfactory results in patients with T1–2 lacrimal gland carcinoma. Disease stage T3 and above was associated with poor outcomes even with post-operative RT, probably due to distant metastasis. Adding neoadjuvant or adjuvant chemotherapy to current treatment strategies may be a suitable choice for this group of patients.

## Data Availability

The datasets used during the current study are available from the corresponding author on reasonable request.
